# Science behind policy: implementing a modern circumference-based body fat equation with a physical fitness threshold is associated with lower musculoskeletal injury risk

**DOI:** 10.1038/s41366-024-01701-5

**Published:** 2025-02-27

**Authors:** Holly L. McClung, P. Matthew Bartlett, Barry A. Spiering, Stephen A. Foulis, Tyler E. Oliver, Leila A. Walker, Vy T. Nguyen, Susan P. Proctor, James P. McClung, Kathryn M. Taylor

**Affiliations:** 1 U.S. Army Research Institute of Environmental Medicine (USARIEM), Natick, MA USA; 2 Oak Ridge Institute for Science and Education (ORISE), Oak Ridge, TN USA

**Keywords:** Weight management, Body mass index, Health policy

## Abstract

**Background:**

Body composition influences physical fitness (PF) and risk of musculoskeletal injury (MSKI). Assessing the relationship between body fat (BF), PF and MSKI risk in a large diverse military population may provide evidence basis informing health-care policies, practices, and programs for military and civilian populations.

**Objective:**

Evaluate the validity of expedient methods to estimate BF (e.g., circumference-based equation (CBE) and bioelectrical impedance analysis (BIA)) and investigate relationships between BF and PF with MSKI risk in a large diverse population.

**Methods:**

Participants were 1904 active-duty Soldiers (643 F) representing Army demographics sex, race/ethnicity (R/E), and age. PF, defined as the most recent Army Combat Fitness Test (ACFT) score and incidence of MSKI, were obtained from Army records. BF was determined by dual-energy x-ray absorptiometry (%BF_DXA_), bioelectrical impedance analysis (%BF_BIA_), and CBE using 3-site (Hodgdon, %BF_HE_) and 1-site (Taylor-McClung, %BF_TM_) equations. Results were stratified by race and sex, to evaluate differences in accuracy of estimated %BF (weighted root mean squared error from %BF_DXA_). Associations of BF and PF with MSKI risk were evaluated with logistic regression.

**Results:**

CBE and BIA underestimated %BF compared to %BF_DXA_. %BF_BIA_ differed from %BF_DXA_ overall and by sex. %BF_TM_ underestimation was uniform across both sex and R/E compared to %BF_DXA_. Mean differences from %BF_DXA_ by sex (M;F) were lower when measured by %BF_TM_ (4.38; 4.59) compared to %BF_HE_ (5.88; 4.39). Individuals had a greater likelihood of MSKI if they failed BF standards (odds ratio 1.32). Scoring ≥ 540 total on ACFT exhibited a 31% (95% CI: 0.52, 0.92) lower MSKI risk during the following 12 months than those with a lower score.

**Conclusions:**

A single-site BF equation (%BF_TM_) maintained similar accuracy across the Soldier population by sex, age, and R/E. Implementing a PF score threshold in lieu of passing Army BF standards was associated with lower MSKI risk.

## Introduction

Body size and composition measures are routinely used to characterize and document changes in an individual’s weight status and to evaluate disease risk by clinicians [1]. Body composition, specifically the ratio of lean-to-fat-mass is highly correlated to physical fitness (PF) [2–4]. Research also links body composition and PF to the incidence of musculoskeletal injury (MSKI) in active populations [5, 6]. In military populations, active young adults, for the most part, a bimodal or J-shaped association between body size and MSKI risk has been observed, with the highest and lowest extremes of body mass index (BMI) at greatest MSKI risk [7, 8]. In the military, lower extremity MSKI incidence is greatest with most common injuries identified as muscle strains, sprains, overuse knee conditions, and stress fractures [8]. A recent systematic review that included both military and civilian populations, found associations between PF, assessed through various measures of both endurance and strength performance and risk of MSKI [9]. Thus, the synergistic relationship between body composition and PF outcomes may provide a functional outcome for use in research and may contribute to injury prevention.

Body composition assessment techniques range in complexity, equipment needed, expense, the requirement for trained technicians, and the selection of technique will depend on the population being assessed and the environment in which the assessment is to be conducted. One laboratory technique, dual energy x-ray absorptiometry (DXA) is a modern technology that is widely accepted and used in the clinical and research fields for measurement of body fat (%BF) and lean mass (LM). Precision of this measurement is better than ±0.5% for BF and ±1.0% for total body bone mineral density (BMD) [10], and as such it is often the criterion method or gold standard, against which precision of other methods is judged. While DXA assessment provides a more detailed understanding of body composition distribution by region and body segment, its expense, lengthy assessment time, large equipment footprint, requirement for trained operators, and exposure to a low level of radiation preclude its use in many environments outside medical treatment facilities and laboratories [11]. Bioelectrical impedance analysis (BIA) is a technology that is used in BF assessment in a variety of settings outside of medical facilities and laboratories due to its much lower cost, smaller equipment footprint, minimal training requirement, and no radiation exposure [12]. Briefly, the BIA method functions based on the principle that electric current flows at different rates through the body depending on the water content of tissues, fat and lean tissue have different water content [13], thus by analyzing the impedance of a current flowing through the body, an estimate of body fat and lean mass can be obtained [14]. Therefore, potential limitations to BIA’s utility for some assessment settings is inability to ensure that those being measured have a normal hydration status and reliability of measurement across sex, race, and ethnicity in a large population [15]. Circumference-based equations (CBE) are a clinically accepted assessment technique to estimate percent body fat (%BF), although inter-rater reliability may hinder use at the research level, they are widely used because of their minimal footprint, low cost, and time efficiency [16].

The U.S. Army is one of the largest, most heterogenous population of active adults to bi-annually track both body composition (by CBE method) and PF to standards as a policy requirement to maintain the health and fitness of Soldiers. Since 1960, the Army has maintained a body composition or maximum weight standard (age- and sex-specific) for its more than one million Soldiers (active-duty, Reserve and National Guard) through a two-step assessment process [17]. Currently, Soldiers who do not meet sex- and age-specific body fat standards then are required to have body fat assessed by manual circumference measurements (two sites for males: neck and abdomen; three sites for females: neck, waist, and hips) at the unit level for estimation by sex-specific CBE [18]. Population-level assessments of a heterogenous sample, in terms of age, sex, race/ethnicity (R/E), and body fatness to compare traditional CBE methods to modern technology body composition assessment techniques and evaluate the association between %BF, PF, and the risk of MSKI are lacking.

The objective of this study was to directly compare different BF measurements against the gold standard %BF_DXA_ (step 1) and to examine the link between BF, PF, and risk of MSKI (step 2) among a representative U.S. Army study population. The goal of this research was to provide a comprehensive approach to future Army policy that applies field efficient and accurate methodology equitable across the diverse Soldier population (accounting for sex, age, and R/E) and ensures accountability for the long-term health and performance of the Solider.

## Methods

### Ethics approval and consent to participate

This study and all research methods were approved by the U.S. Army Medical Research and Development Command Human Institutional Review Board (Fort Detrick, MD). Investigators adhered to the policies regarding the protection of human subjects as prescribed in Army Regulation 70-25, and the research was conducted in adherence with the provisions of 32 CFR Part 219. Data collection took place October 2021 to July 2022 at three Army installations: Fort Liberty, NC; Fort Gregg-Adams, VA; and at the U.S. Military Academy, West Point, NY (cadets were excluded). All participants provided written informed consent. Exclusion criteria included current pregnancy and large amounts of metal in the body that would impact body scanning procedures.

### Study design and participants

The study population was a representative sample of 1904 (643 Females; 1259 Males) active-duty Soldiers (Supplementary Table 1). The population was selected using validated methodology similar to the National Health and Nutrition Examination Survey (NHANES) in order to produce a study population that was representative of the U.S. Army demographic distributions of sex, race/ethnicity (R/E, American Indian or Alaskan Native, Asian/Pacific Islander, Black non-Hispanic, Hispanic, White non-Hispanic, and other) and age category (17–20, 21–27, 28–39, >40-years old) that existed in the Army in December 2020 [19]. Women and groups comprising less than 10% of the populations (American Indian or Alaskan Natives, Asians and those >40 years old) were oversampled to better evaluate between groups differences in these smaller populations.

### Data collection

#### Demographic and physical performance data

Participants provided baseline demographic and military information by consenting to provide their Department of Defense (DoD) identification number to allow access to current Army Combat Fitness Test (ACFT) scores. At the time of data collection, the ACFT included six events: 3 repetition maximum deadlift, standing power throw, hand-release push-up, sprint-drag-carry, 2-mile run and the leg tuck. The ACFT events were scored according to age and sex adjusted standards (maximum of 100 points/event and maximum cumulative total score of 600). In March 2022, the leg tuck was dropped from the ACFT and replaced with a timed plank [20]. To account for this change within analyses containing the ACFT, scores for the leg tuck were dropped from the total ACFT scores and it was assumed that all passed the timed plank at the minimum score needed for each threshold score analysis. MSKI data was accessed through a data repository maintained by the US Army Research Institute of Environmental Medicine, the Soldier Performance Health and Readiness database. For this study, MSKI was defined as any injury that resulted in lost or limited duty time resulting in a medically documented profile in the one year following the participant’s body composition assessment. Average duty time lost for the study population was 57 ± 58 days (mean ± SD; range 2–365 days).

#### Body composition assessment

Participants were asked to come to data collection site fully hydrated, with at least 12 h since the last exercise bout and three hours since their last meal. Anthropometric measurements were made in lightweight shirt, shorts, and sports bra (for women) with stocking feet. Standing height was measured using a stadiometer (model 217, SECA, Chino, CA) and body mass was measured using a calibrated electronic scale (model DS6150, Doran, Batavia, IL).

Body composition was determined using four techniques: dual-energy x-ray absorptiometry (%BF_DXA_, GE Lunar Prodigy Advanced, GE Healthcare, Madison, WI), bioelectrical impedance analysis (%BF_BIA_, InBody 770, InBody, Cerritos, CA) and CBE. Female participants produced a negative pregnancy test prior to scanning procedures. For CBE, manual circumference for both men and women was measured at the neck, waist, abdomen, and hips in triplicate. The measurements were conducted by trained research staff using a calibrated fiberglass tape measure and recorded to the nearest 0.5 inch, body weight (BW) to the nearest pound, and height to the nearest 0.5 inch. The circumference measured was used to estimate %BF using two separate, sex-specific equations (more details on these equations are provided below) [18, 21–23].

Hodgdon equations from the 2019 Army Regulation (%BF_HE_) [18, 21]:

$${\rm{Female}}\!:\left[163.205{\rm{x}}\; {\rm{Log}}10\left({\rm{waist}}+{\rm{hip}}-{\rm{neck}}\right)\right]-\left[97.684\,{\rm{x}}{\rm{Log}}10\left({\rm{height}}\right)\right]-78.387$$

$${\rm{Male}}\!:\left[86.010\,{\rm{x}}{\rm{Log}}10\left({\rm{abdomen}}-{\rm{neck}}\right)\right]-[70.041\,{\rm{x}}{\rm{Log}}10({\rm{height}})]+36.76$$

Taylor-McClung equations updated for use in 2023 Army Regulation (%BF_TM_) [22, 23]:

$${\rm{Female}}\!:-9.15-(0.015{\rm{x}}\; {\rm{BW}})+(1.27{\rm{x}}\; {\rm{abdomen}})$$

$${\rm{Male}}\!:=-26.97-(0.12{\rm{x}}\; {\rm{BW}})+(1.99{\rm{x}}\; {\rm{abdomen}})$$

The Taylor-McClung equation [22] was developed as a simpler CBE for estimation of %BF with error equitable across sex, age, and R/E as compared to %BF_DXA_, a research standard of measure [24]. The in vivo coefficient of variation in an external population for soft tissue and %BF_DXA_ was 0.4 to 1.0% [25].

The TM equation was further examined herein to determine whether a built-in offset would more closely meet the needs of the Army while improving the %BF estimation to accurately categorize individuals as meeting the Army %BF standards. To do this, a 1% and 2.5% offset was built into the base TM Equation [22]. This allowed the %BF of all individuals to be slightly underestimated with the goal of reducing error where an individual would be falsely failed by the CBE method. In other words when there is error in categorizing an individual for meeting the Army %BF standards, it would be in favor of the Soldier.

### Statistical analysis

The population was weighted to provide results that would represent a population with the same distribution of age, R/E, and sex as the current U.S. Army. This was done by classifying each individual into a combined age, R/E, and sex category. This weight was calculated using the following equation:

$$\frac{\Pr ({Population\; Race})\times \Pr \left({Population\; Sex}\right)\times \Pr ({Population\; age})}{\Pr ({Sample\; Race})\times \Pr \left({Sample\; Sex}\right)\times \Pr ({Sample\; age})}$$

where each individual was assigned, a weight based on the proportion (Pr) of their specific age, R/E and sex category in both the study sample and in the entire Army. This weighting redistributed the distribution of age, R/E, and sex within the study population to be equal to that of the Army.

#### Step 1 – BF methods comparison

Weighted proportions were estimated for the pass and fail rates and the false pass and false fail rates for each of the circumferences-based equations using the AR 600-9 body composition standards. A false pass was indicated when an individual passed the AR 600-9 based on the CBE but should have failed if the %BF_DXA_ was used. A false failure was indicated when an individual failed the AR 600-9 using the CBE but would have passed if %BF_DXA_ was used. To estimate accuracy in the %BF as estimated by the CBEs and BIA, weighted root mean squared error was calculated using %BF_DXA_ as the gold standard. Results were presented by R/E and sex, to evaluate potential differences in accuracy of the estimated %BF. To evaluate accuracy of the prediction equations to correctly identify passing or failing AR 600-9, area under the curve for the receiver operating characteristic curve (ROC AUC) was estimated using a logistic regression model.

#### Step 2 – examination of link with performance and injury

To evaluate the associations of body composition and PF on MSKI risk, logistic regression models were used. Separate models were run to evaluate how passing the Army %BF standard and how %BF as continuous variables affected MSKI risk. Additionally, models were built to evaluate how achieving three different thresholds of scores on the ACFT effected injury risk. The three ACFT thresholds evaluated were: (1) scoring above 60 points on all six events or passing the current ACFT standard (ACFT_pass/fail_), (2) scoring above 80 points on all six events (ACFT_80_), (3) scoring above 90 points on all six events (ACFT_90_), and (4) scoring at least 80 points on all six events while simultaneously scoring a total score above 540 points, i.e., >80 for enough events to accumulate 40 additional points, (ACFT_540_). ACFT models were adjusted for age and sex to account for the age and sex standardized scoring system of the ACFT. The goal of these models was to identify a threshold score on the ACFT that significantly protected against injury and had similar pass rates for both men and women. Once an ideal ACFT threshold was identified, individuals were classified as meeting that standard or not. A logistic regression model with an interaction term between meeting the identified ACFT threshold score and continuous %BF_DXA_ was performed to evaluate if meeting the ACFT threshold modifies the association of %BF on MSKI risk. Approximately 20% of the participant pool had missing ACFT data. Sensitivity analysis was conducted, and with no bias was associated with missing ACFT data. To include all observations in the analysis, multiple imputation with 5 imputations were used for analyses conducted with ACFT data.

Statistical analyses were conducted using R (v4.2.1; R Core Team 2022) and SAS statistical software (Version 9.4, SAS Institute Inc Cary, NC, USA 2023).

## Results

### Population characteristics

The study population was sampled and weighted to represent the 2021 Army active-duty population across sex, age, R/E, and military rank (Supplementary Table 1). Body size and composition measures varied across sex (Table 1). Compared to women, men were taller (mean difference ± SE; 12.65 ± 0.34 cm), had a greater body mass (5.96 ± 0.63 kg), and body mass index (BMI) (1.58 ± 0.18 kg·m-2). Men were leaner (%BF_DXA_ −8.63 ± 0.30) had greater fat-free mass (17.62 ± 0.41 kg) and greater bone mineral density (0.09 ± 0.01 g/cm) than women (*p* < 0.001).

**Table 1:** Population weighted means for body size composition and measurements stratified by sex.

	Total	Men	Women
	*n* = 1902	*n* = 1259	*n* = 643
Height, cm	174.8 ± 0.2	176.8 ± 0.2	163.8 ± 0.3
Total body mass, kg	84.3 ± 0.3	86.9 ± 0.4	70.3 ± 0.4
BMI, kg/m^2^	27.5 ± 0.1	27.8 ± 0.1	26.1 ± 0.1
Body Composition by DXA			
Fat mass, kg	22.1 ± 0.2	21.8 ± 0.2	23.4 ± 0.3
Fat-free mass, kg	59.1 ± 0.2	61.9 ± 0.2	43.7 ± 0.3
Bone mass, kg	3.1 ± 0.0	3.2 ± 0.0	2.5 ± 0.0
BMD, g/cm	1.3 ± 0.0	1.4 ± 0.0	1.3 ± 0.0
% BF_DXA_	25.9 ± 0.2	24.6 ± 0.2	33.1 ± 0.2
% BF_HE_	21.7 ± 0.1	19.9 ± 0.2	31.4 ± 0.2
% BF_TM_	25.7 ± 0.1	24.3 ± 0.1	33.2 ± 0.2
% BF_BIA_	24.6 ± 0.2	23.4 ± 0.2	31.2 ± 0.2

Data is weighted Mean ± SE.

*BMI* body mass index, *DXA* dual-energy x-ray absorptiometry, *BMD* bone mineral density, *HE* Hodgdon equations [19], *TM* Taylor-McClung equations [21], *BIA* bioelectrical impedance analysis.

### Body composition method outcomes

Estimation of %BF by both CBEs (%BF_HE_ and %BF_TM_) and BIA (%BF_BIA_) methods differed from the ‘true’ measure of standard (%BF_DXA_) overall and by sex. Overall, mean differences (+/− SE) estimated by %BF_BIA_ were most similar to %BF_DXA_ (−1.27 ± 0.10%)_,_ followed by %BF_TM_ (−2.57 ± 0.09%) and %BF_HE_ (−4.39 ± 0.07%). The %BF_BIA_ estimation had the greatest variability in measurement overall and across sex; estimation by %BF_TM_ provided the least variability across sex.

**Fig. 1:**
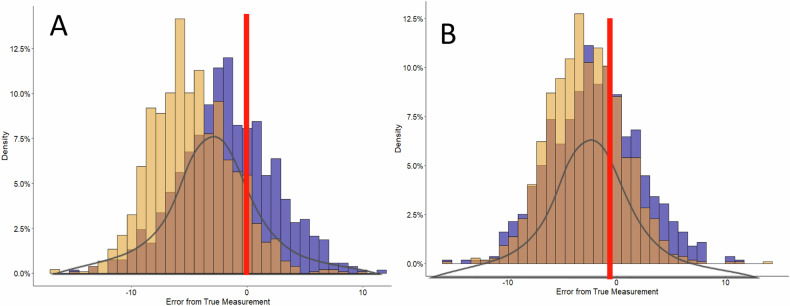
Distribution of error by population density for estimation of percent body fat (%BF) by circumference-based methods (CBE) as compared to the ‘True Measurement’ by the dual-energy x-ray absorptiometry. **A** %BF estimated by the Hodgdon equations (%BF_HE_) **B** %BF estimated by the Taylor-McClung equations (%BF_TM_). Color denotes population breakout: brown, overlap of distribution for males and females; yellow, male only distribution; purple, female only distribution.

Offset options to the TM equation and potential impacts on the study population are listed in Table 2 with ROC AUC prediction performance in Supplementary Table 2. A 1% offset improved the overall accuracy in BF failures with approximately equivalent false fail rates for men and women and increased the proportion of both male and female Soldiers who falsely passed. However, integration of a 2.5% offset (%BF_TM_) maintained the improved accuracy of the %BF estimation compared to the %BF_HE_, and further equalized the distribution of inaccuracies in pass/fail rates of standards across sex. Comparing use of CBE for %BF estimation, both %BF_HE_ and %BF_TM_ underestimated %BF in favor of the Soldier as compared to %BF_DXA_ (Table 2 and Fig. 1). This distribution of error after use of %BF_TM_ with 2.5% offset (Fig. 1B) for %BF estimation was more equitable for men and women across R/E categories compared to use of %BF_HE_ (Fig. 1A).

**Table 2:** The function of Taylor-McClung circumference-based equations (CBE) with offsets and the addition of ACFT performance built into Army body fat policy to accurately classify body fat (%BF) by Army standards.

Measurement	Meet BF Stds	Fail BF Stds	Accurately Pass Stds^a^ (True Positive)	Accurately Fail Std^a^ (True Negative)	Inaccurately Pass Stds^a^ (False Positive)	Inaccurately Fail Std^a^ (False Negative)
%BF_DXA_	43.06%	56.94%	-	-	-	-
Male	42.00%	58.00%	-	-	-	-
Female	49.07%	50.95%	-	-	-	-
%BF_HE_	72.34%	27.65%	41.79%	26.35%	30.55%	1.30%
Male	74.08%	25.92%	41.36%	25.28%	32.72%	0.64%
Female	62.84%	37.16%	44.20%	32.25%	18.64%	4.91%
%BF_TM_	53.71%	46.29%	37.05%	40.25%	16.66%	6.04%
Male	46.97%	53.03%	30.14%	47.24%	16.83%	5.79%
Female	57.42%	42.58%	41.70%	35.16%	15.72%	7.42%
%BF_TM_ + 1% offset	58.35%	41.65%	39.03%	37.57%	19.32%	4.08%
Male	57.02%	42.98%	37.79%	38.77%	19.23%	4.21%
Female	65.58%	34.41%	45.77%	31.06%	19.81%	3.35%
%BF_TM_ + 2.5% offset^b^	66.81%	33.19%	41.53%	31.66%	25.28%	1.53%
Male	66.02%	33.97%	40.58%	32.56%	25.44%	1.41%
Female	71.40%	28.60%	46.98%	26.46%	24.42%	2.14%
%BF_TM_ + 2.5% offset^b^ + ACFT_pass/fail_	93.51%	6.49%	42.90%	6.33%	50.61%	0.16%
Male	93.94%	6.06%	41.82%	5.92%	52.12%	0.14%
Female	91.15%	8.85%	42.36%	8.60%	48.79%	0.25%
%BF_TM_ + 2.5% offset^b^ + ACFT_80_	75.63%	24.37%	42.34%	23.65%	33.29%	0.72%
Male	74.31%	25.69%	41.25%	24.98%	33.06%	0.71%
Female	82.82%	17.18%	48.32%	16.41%	34.50%	0.77%
%BF_TM_ + 2.5% offset^b^ + ACFT_90_	69.22%	30.78%	41.74%	29.47%	27.48%	1.31%
Male	68.09%	31.91%	40.77%	30.72%	27.32%	1.19%
Female	75.38%	24.57%	47.05%	22.58%	28.33%	1.99%
%BF_TM_ + 2.5% offset^b^ + ACFT_540_	71.27%	28.72%	42.07%	27.74%	29.20%	0.98%
Male	70.14%	29.86%	41.01%	28.91%	29.13%	0.95%
Female	77.48%	22.51%	47.88%	21.34%	29.60%	1.17%

*CBE* circumference-based equation, *BF* body fat, *DXA* dual-energy x-ray absorptiometry, *HE* Hodgdon equations [18, 21], *TM* Taylor-McClung equations [22].

^a^Accuracy determined by % BF_DXA_ measurement as ‘true measurement’.

^b^CBE used in 2023 Army Policy [23].

Overall, by sex and R/E, %BF was underestimated by all methods (%BF_HE_, %BF_TM_, and %BF_BIA_) as compared to %BF_DXA_. Estimation of %BF methods varied in mean difference from %BF_DXA_ as examined across sex and R/E categories (Table 3). As was previously reported, the average measurement error in %BF or bias (95% CI) varied by sex for %BF_HE_ with females experiencing on average −1.69% (95% CI: −9.69, 6.25%) error and males experiencing −4.79% (95% CI: −11.50, 1.89%) error [22]. This is compared to the %BF_TM_ with 1% and 2.5% offsets where both men and women experience approximately similar level of biases with greatest difference of 0.77% bias for men (Bias: −2.81, 95% CI: −9.41, 3.79) and women (bias: −2.04, 95% CI: −10.10, 6.04) with the 2.5% offset (Supplementary Fig. 1). %BF_TM_ differences were significant (*p* < 0.001) across both sex and all R/E as compared to %BF_DXA_ with an average underestimation of ~3%. %BF_BIA_ differences from the %BF_DXA_ were significant only for males across R/E categories (Table 3).

**Table 3:** Weighted mean measurements and differences (bias) between body fat assessment methods compared to DXA.

Race/Ethnicity	Am Indian/AK Native	Asian/PI	Black	Hispanic	White
	**Female**				
DXA	33.95 ± 1.35	34.33 ± 0.75	33.49 ± 0.40	34.06 ± 0.49	31.85 ± 0.40
CBE_HE_	−3.05 (−6.51, 0.42)	−3.01 (−4.06, −1.97)	−1.16 (−1.73, −0.60)	−1.92 (−2.68, −1.16)	−1.58 (−2.08, −1.08)
CBE_TM_	−3.22 (−6.42, −0.02)	−3.67 (−4.70, −2.64)	−2.44 (−3.00, −1.89)	−2.55 (−3.24, −1.85)	−0.94 (−1.47, −0.41)
BIA	−2.39 (−5.79, 1.00)	−2.72 (−3.69, −1.75)	−2.05 (−2.57, −1.53)	−2.19 (−2.90, −1.48)	−1.34 (−1.82, −0.86)
	**Male**				
DXA	26.96 ± 1.73	23.92 ± 0.44	22.76 ± 0.43	25.79 ± 0.38	24.85 ± 0.24
CBE_HE_	−5.72 (−6.71, −4.75)	−4.86 (−5.42, −4.29)	−4.56 (−4.97, −4.14)	−5.02 (−5.43, −4.60)	−4.54 (−4.82, −4.27)
CBE_TM_	−3.82 (−5.43, −2.21)	−2.96 (−3.50, −2.42)	−2.86 (−3.29, −2.42)	−3.29 (−3.72, −2.86)	−2.46 (−2.74, −2.19)
BIA	−1.26 (−3.63, 1.10)	−2.94 (−3.56, −2.33)	−0.68 (−1.19, −0.18)	−1.73 (−2.25, −1.20)	−1.00 (−1.32, −0.69)

Mean ± SE. Differences as 95% CI.

*DXA* dual-energy x-ray absorptiometry, *CBE* circumference-based equation, *HE* Hodgdon equations [18, 21], *TM* Taylor-McClung equations used in Army Regulation update 2023 [22], *BIA* bioelectrical impedance analysis.

### Body composition outcomes by physical performance, injury

**Fig. 2:**
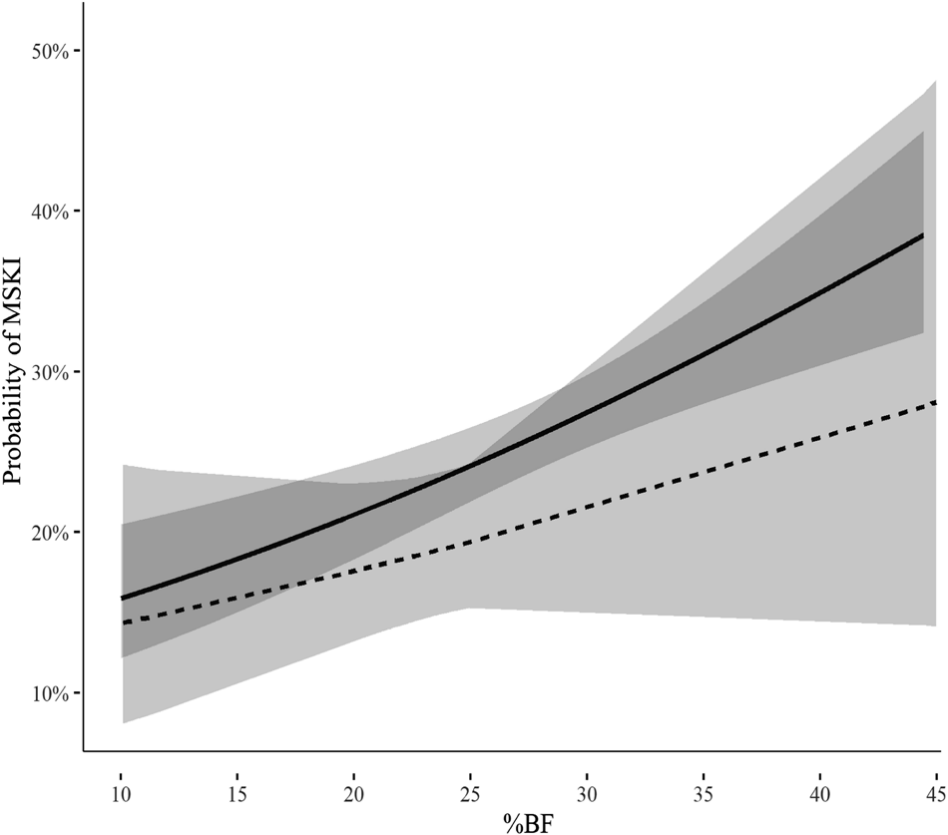
The probability of having an MSKI related to %BF_DXA_ stratified by passing the ACFT_540_ threshold. The grey area represents the 95% confidence intervals for the probability (black lines) at each %BF_DXA_. The dotted line is the estimated probability of MSKI in those who passed the ACFT at the ACFT_540_ threshold. The solid line is the estimated probability of MSKI in those who did not pass the ACFT at the ACFT_540_ threshold.

Individuals who passed the Army %BF standards using the %BF_DXA_, compared to those who failed, had a 24.9% reduction (*p* = 0.01) in the odds of a developing a duty-limiting MSKI in the year after their assessment. When %BF_DXA_ was treated continuously in the model, increasing %BF_DXA_ was associated with increased injury (Odds ratio: 1.04, 95% CI: [1.03, 1.04], *p* < 0.001). Evaluating how incorporation of the 4 ACFT thresholds would impact the accuracy of meeting the Army %BF standards, ACFT_pass/fail_ had the lowest false fail rate but resulted in an ~25% increase in the false pass rate (Table 2). ACFT_90_ and ACFT_540_ had similar false pass rates, while ACFT_540_ reduced the false failure rate to ~1%. Overall, for every point increase on the ACFT there was 0.05% (95% CI: −0.07, −0.02; *p* < 0.001) reduction in %BF. Among the four thresholds evaluated for the ACFT, scoring above ACFT_80_ and scoring above ACFT_540_ significantly reduced the development of an MSKI by 25% and 31%, respectively (Table 4). The improvement in each equation to predict passing Army %BF standards with inclusion of the ACFT thresholds, demonstrated very good predictive (>0.80) values across all ACFT thresholds (Supplementary Table 2). The ACFT_540_was selected as the ideal threshold due to its higher MSKI reduction. While independent models demonstrated that achieving an ACFT_540_ or %BF was protective against MSKI, in model assessing the potential effect modification of passing the ACFT_540_ on the risk of MSKI associated with increasing %BF_DXA_ the interaction term did not reach significance (*p* = 0.15). While it appears that the ACFT_540_ attenuates the risk of increased MSKI associated with increasing %BF (Fig. 2), the lack of statistical significance may be driven by high correlation between those who meet the ACFT_540_ and those who met Army %BF standards where 75% of those who meet the ACFT_540_ also met the Army %BF standard.

**Table 4:** The odds of MSKI associated with scoring above Army physical fitness threshold score.

Threshold Score	Scored < Threshold		Scored ≥ Threshold		Effect Estimate	95% Confidence Intervals	*p*-value
	M	F	M	F			
ACFT_pass/fail_	12.70%	21.71%	87.30%	78.29%	0.94	(0.68, 1.29)	0.69
ACFT_80_	78.14%	78.28%	21.86%	21.72%	0.75	(0.56, 0.99)	0.048
ACFT_90_	94.08%	89.93%	5.92%	10.07%	0.55	(0.30, 1.00)	0.051
ACFT_540_	80.78%	78.28%	19.22%	21.72%	0.69	(0.52, 0.92)	0.011

*ACFT* Army Combat Fitness Test (6 event test; max 100 points/event with total max score 600 points), *MSKI* musculoskeletal injury, *ACFT*_*pass/fail*_ pass the current ACFT standard, *ACFT*_*80*_ score above 80 points on each event, *ACFT*_*90*_ score above 90 points on each event, *ACFT*_*540*_ scored above 80 points on each event and 540 points on the total score.

## Discussion

This study is the first to compare accuracy and application of methods to assess body composition, both traditional and modern, in a large, diverse, population. In addition, mathematical modeling was used to allow for the dynamic integration of PF thresholds and MSKI incidence for a holistic approach to recommendations for body composition standards in active populations. Outcomes from this work propose the use of a newly developed CBE equation with an Army-specific 2.5% offset to more accurately classify Soldiers as meeting Army-specific body fat requirements and needs to maintain a healthy force and have formed the basis for the 2023 Army body composition standard policy [23].

A strength of this study is the sample population; to our knowledge it is the largest, most modern, and heterogenous sample, stratified by age, R/E, and body fatness used to compare body composition methods. Like the U.S. population, the Army has a broad range of demographics leading to many different body shapes and sizes. Accuracy of body composition methods and equations have been shown to be population-specific [26], and often correlated for use within a specific sex, age range, R/E and/or level of fitness [16, 27]. However, the Army is required to standardize body composition methodology by using one method to assess all Soldiers across all ages, R/E, and fitness with equal bias, and the highest accuracy possible.

Body composition methodology and technologies vary in accuracy and applications. The two methods selected, CBE and BIA, for investigation in this study have similar ranges of accuracy as reported in the literature [16, 28]. The methods examined herein utilize two versions of the CBE method (range of accuracy) with use of manual measurements, traditional HE (−17.16 to 10.99) and modern TM [22] (−12.91 to 13.50; with Army-specific offset 15.36 to 11.39), and use of the BIA technology (15.10 to 13.65). Assessment methods were selected for study inclusion based on accuracy as compared with the current method in use (%BF_HE_) and feasibility of use by the Army. Although %BF_BIA_ method appeared to have greater overall accuracy in measurement as compared to the %BF_DXA_, cost and the need to monitor hydration status may be problematic for some Army organizations [15]. Individuals participating in this study were asked to arrive hydrated, fasted (~3 h post-meal) and 12-h post-exercise, and it is not likely that these conditions will be achieved with wide Army use of BIA. Further, the BIA literature requires validation in large demographically diverse populations to be considered for use in large-scale Army policy updates. CBE remains the most cost- and time-efficient methodology with feasible application in any environment or setting. Therefore, based on the selection factors defined, CBE continues to be the method of choice for clinicians, in research, and for the Army.

Use of the CBE used to estimate %BF by the Army prior to 2023 (%BF_HE_) functioned well at the population level in determining Army-specific BF standard cut points outlined in Army policy (AR 600-9) [22], however across sex, differences in accuracy existed (Table 2). Overall, %BF_HE_ underestimates %BF, to a greater degree for males, therefore allowing a disproportional number of male Soldiers to inaccurately fall below the BF standards as compared to the female Soldier population. The %BF_TM_ with 2.5% offset improved the accuracy of BF estimation (compared to %BF_DXA_) and more equally distributed error in pass/fail rates for the Army BF standards across sex and R/E compared to %BF_HE_ Army standard BF cut points (Fig. 2 and Tables 3–4). In addition, compared to the use of %BF_HE_, the %BF_TM_ decreases time and training since just one anatomical landmark across sex is required to be measured for BF estimation. However, the TM equation underestimates the Soldier population passing the Army BF standards because the mean error for the equation was zero allowing for individuals to be both over- and underestimated for %BF compared to %BF_HE_. Integration of a PF threshold associated with healthy %BF is an additional layer for consideration in body composition policy.

Body composition, PF, and MSKI are synergistic in maintaining physical- and job-related goals and standards. Healthy body composition (ratio of %BF to %LM) is associated with improved physical performance in athletes and Soldiers [29, 30]. As has been reported in college and professional athletes [31], MSKI causes the greatest lost training time and, in Soldier populations, the greatest restrictions to performance or work [32]. In this investigation, mathematical modeling demonstrated the effect of a PF threshold, the ACFT_540_ had a similar protective effect for MSKI risk (with a reduction of 31%) as passing Army %BF standards [23]. Defining a PF threshold for use alongside %BF_TM_ further improved the accuracy by reducing the number of individuals that would falsely fail the Army-specific BF standards to near zero (0.7% for women and 0.5% for men). This triad of healthy lifestyle factors together reinforces the long-term health of the individual by motiving improved PF with a performance goal linked to healthy body composition, both in turn minimizing MSKI incidence.

A limitation of this study is that the %BF_TM_ has only been validated in an Army population [22]. Future research should look to validate the TM equation (with or without the 2.5% offset) in a diverse, non-Army population to determine if the method is more generalizable. Serial comparison of %BF_TM_ and PF threshold across a modern U.S. active, adult population could provide functional outcomes for use in preventative health care and monitoring. Future adjustments to the TM equation may be warranted as the Army population demographics shift or Army leadership looks to align body composition to a different (higher) PF score or a change in MSKI rate is defined. Additionally, further research is needed to align the Army-specific performance outcomes to more general, civilian-applicable activities and levels.

In conclusion, a novel single-site BF equation (%BF_TM_) maintained accuracy by sex, age, and R/E as compared to traditional CBE and BIA methods. Implementing a PF threshold to Army-specific BF standards lowered MSKI risk. This research is the first to correlate PF and healthy body composition to MSKI outcomes in a diverse and physically active adult population. Incorporating, PF into body composition assessments will help promote fitness as a primary focus, which could have positive impacts on body composition and health outcomes both short- and long-term to benefit the individual. The impact of cumulative healthy outcomes shifts the focus to a more holistic approach of healthy body size and composition to a paradigm that supports peak physical performance and reduces injuries.

## Disclaimer

The investigators have adhered to the policies for protection of human volunteers as prescribed in Army Regulation 70–25, and the research was conducted in adherence with the provisions of 32 CFR Part 219. 2. Citations of commercial organizations and trade names in this paper do not constitute an official Department of the Army endorsement or approval of the products or services of these organizations. This research was supported in part by an appointment of a researcher to the Department of Defense (DOD) Research Participation Program administered by the Oak Ridge Institute for Science and Education (ORISE) through an interagency agreement between the U.S. Department of Energy (DOE) and the DOD. The opinions or assertions contained herein are the private views of the authors and are not to be construed as official or as reflecting the views of the Army or the DOD, DOE, or ORAU/ORISE.

## Supplementary information

Supplemental Materials

## Data Availability

Data described in the manuscript, code book, and analytic code will not be made available because due release of data is considered PII and lack of consent by study participants for dissemination beyond immediate study use and sensitivity of the data with potential risk to national security.
