# Supplementary material for: Science behind policy: implementing a modern circumference-based body fat equation with a physical fitness threshold is associated with lower musculoskeletal injury risk
Source: Int J Obes (Lond). 2025 Feb 27;49(4):723–30. doi: 10.1038/s41366-024-01701-5 (PMC11999870; doi:10.1038/s41366-024-01701-5)
Supplement: Supplementary file 1 — Supplemental Materials [file 41366_2024_1701_MOESM1_ESM.pdf]

Figure Legend

Supplemental Figure 1. Bland Altman plots stratified by sex comparing percent body fat measured by DXA (%BF_{DXA}) to the Taylor-McClung equations (%BF_{TM}) with a 1% (Panels A, B) and 2% (Panels C, D) offset. The Bland Altman plots for with no offset and the Hodgon equation (%BF_{HE}) are previously published [22]. The solid black line represents where there is no difference between the equation and %BF_{DXA}. Red dots (Panels A, C) represent female and blue dots (Panels B, D) represent male participants. The black slope line represents the relationship between the difference in the two measurements and the mean of the measurements.

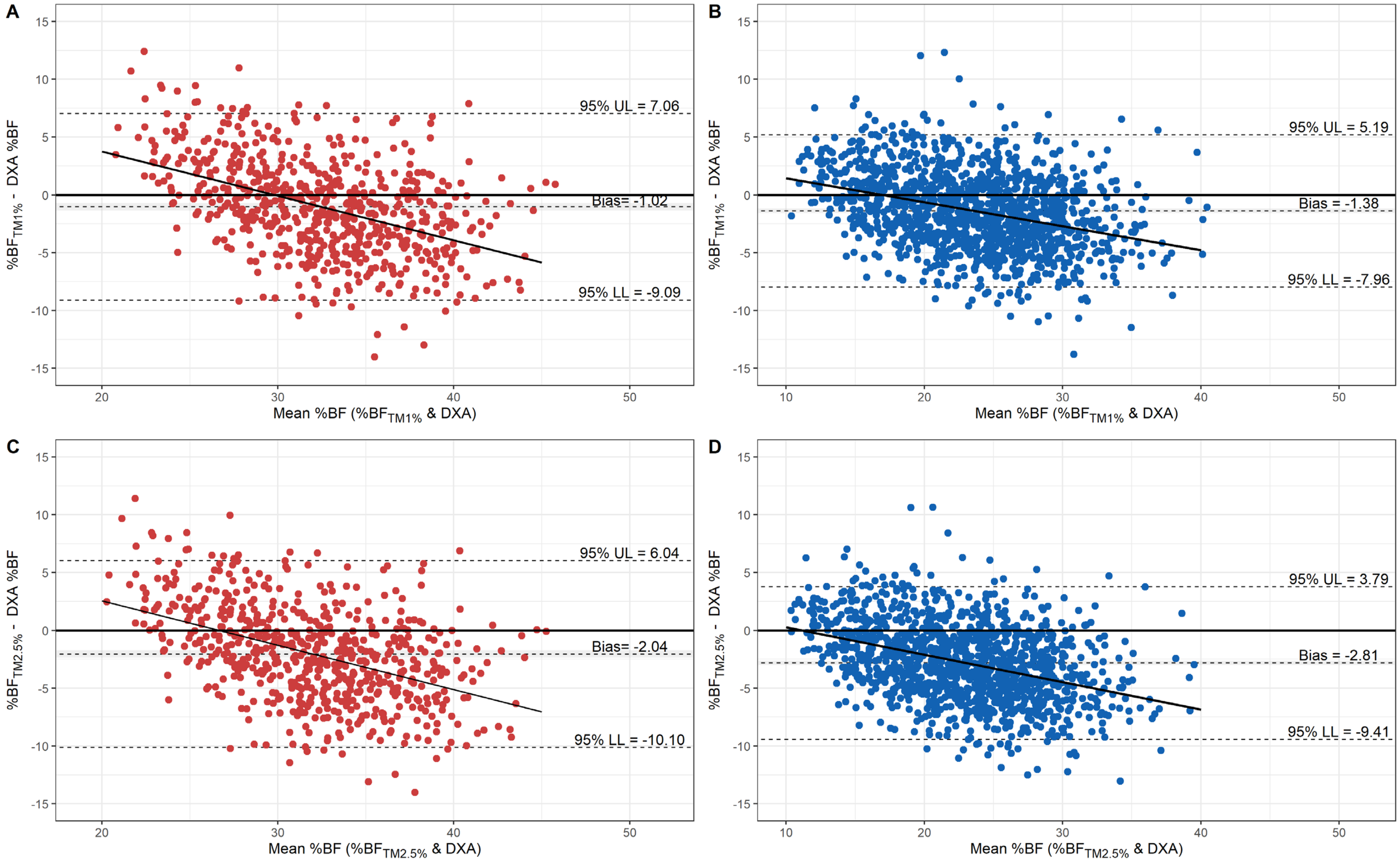
A
B
C
D
95% UL = 7.06
Bias= -1.02
95% LL = -9.09
95% UL = 5.19
Bias = -1.38
95% LL = -7.96
95% UL = 6.04
Bias= -2.04
95% LL = -10.10
95% UL = 3.79
Bias = -2.81
95% LL = -9.41
$\%BF_{TM1\%}$ - DXA %BF
$\%BF_{TM2.5\%}$ - DXA %BF
Mean %BF ($\%BF_{TM1\%}$ & DXA)
Mean %BF ($\%BF_{TM2.5\%}$ & DXA)

Supplemental Table 1. Study population demographics

	% of Army* (2021 Demographics)	Study Population		
		Total	Men	Women
**Sex**		n=1904	n=1261	n=643
Female	16%	33.8%		
Male	84%	66.2%		
**Race/ethnicity n(%)**				
Am. Indian or Alaskan Native	1%	24 (1.3%)	15 (1.2%)	9 (1.6%)
Asian or Pacific Islander	7%	198 (10.4%)	147 (11.7%)	51 (7.9%)
Black, not Hispanic	21%	433 (22.8%)	235 (18.7%)	198 (30.8%)
Hispanic	17%	372 (19.6%)	236 (18.8%)	136 (21.2%)
White, not Hispanic	54%	859 (45.1%)	620 (49.2%)	239 (37.2%)
Other	1%	18 (0.9%)	8 (0.6%)	10 (1.6%)
**Age category n(%)**				
17-20	41%	201 (10.6%)	126 (10.0%)	75 (11.7%)
21-27	40%	820 (43.0%)	540 (42.8%)	280 (43.6%)
28-39	18%	705 (37.1%)	468 (37.1%)	237 (36.9%)
>40	1%	178 (9.4%)	127 (10.1%)	51 (7.9%)
**Rank n(%)**				
Enlisted	82%	1432 (75.1%)	975 (77.3%)	457 (71.1%)
Officer	18%	472 (24.8%)	286 (22.7%)	186 (28.9%)

*Active duty Army Soldier population

Supplemental Table 2. Performance of body fat prediction equations to predict pass/fall of the Army body fat standards at various fitness thresholds.

	ROC AUC (95% CI)	
**Model**	**M**	**F**
CBE_{HE}	0.896 (0.878, 0.913)	0.869 (0.842, 0.896)
CBE_{TM}	0.892 (0.875, 0.910)	0.829 (0.799, 0.860)
CBE_{TM} + $ACFT_{Pass/False}$	0.892 (0.872, 0.911)	0.821 (0.783, 0.859)
CBE_{TM} + $ACFT_{80}$	0.903 (0.885, 0.921)	0.857 (0.823, 0.891)
CBE_{TM} + $ACFT_{90}$	0.905 (0.887, 0.923)	0.829 (0.792, 0.867)
CBE_{TM} + $ACFT_{540}$	0.905 (0.889, 0.922)	0.857 (0.822, 0.891)

ROC, receiver operating characterisitc; AUC, area under the curve; 95% CI, 95% confidence intervals; CBE, circumference-based equation; HE, Hodgdon equations[18, 21]; TM, Taylor-McClung equations[22]; ACFT, Army Combat Fitness Test (6 event test; max 100 points/event with total max score 600 points); $ACFT_{pass/fail}$: pass the current ACFT standard, $ACFT_{80}$: score above 80 points on each event, $ACFT_{90}$: score above 90 points on each event, $ACFT_{540}$: scored above 80 points on each event and 540 points on the total score.
